# New insight into the photocatalytic degradation of organic pollutant over BiVO_4_/SiO_2_/GO nanocomposite

**DOI:** 10.1038/s41598-021-84323-5

**Published:** 2021-02-25

**Authors:** Dang Trung Tri Trinh, Duangdao Channei, Auppatham Nakaruk, Wilawan Khanitchaidecha

**Affiliations:** 1 Department of Civil Engineering, Faculty of Engineering, Naresuan University, Phitsanulok, 65000 Thailand; 2 Centre of Excellence for Innovation and Technology for Water Treatment, Naresuan University, Phitsanulok, 65000 Thailand; 3 Department of Chemistry, Faculty of Science, Naresuan University, Phitsanulok, 65000 Thailand; 4 Department of Industrial Engineering, Faculty of Engineering, Naresuan University, Phitsanulok, 65000 Thailand

**Keywords:** Materials science, Nanoscience and technology

## Abstract

The nanocomposite of BiVO_4_-based material has been synthesized by one-step solvent method. The morphological, physical, chemical properties of the nanocomposite have been investigated. The results revealed that the surface area of BiVO_4_, BiVO_4_/SiO_2_ and BiVO_4_/SiO_2_/GO was 11.13, 28.47 and 43.93 m^2^/g, respectively. The structural test by XRD proved that the nanocomposites were monoclinic phase of bismuth vanadate. Adsorption and photocatalytic degradation were two main mechanisms that strongly related to pollutant removal efficiency (i.e., methylene blue and phenol). The BiVO_4_/SiO_2_/GO nanocomposite obtained the greatest MB removal efficiency due to its high adsorption ability from high surface area, whereas the photocatalytic degradation was insignificant mechanism. In contrast, the relatively low adsorption ability of BiVO_4_/SiO_2_/GO nanocomposite was observed when the pollutant was phenol due to negative charge and high stability of phenoxide ions, then the photocatalytic degradation became the main mechanism for phenol removal. The phenol removal efficiency reached approximately 70% in 6 h with H_2_O_2_ assistance. The combination of SiO_2_ and GO improved the surface property of BiVO_4_-based photocatalyst, however the excessive combination ratio generated the excellent adsorbent material rather than the photocatalyst. Hence, the optimal combination ratio is essential to archive the greatest nanocomposite for photocatalytic application.

## Introduction

In recent years, the water pollution has been causing serious problems influencing on the environmental crisis and the human health due to the release of organic wastewater from the rapid development of industries^1,2^. Many technologies have been used to treat and reuse water source such as biological, chemical and physical processes to control pollutants leading to the sustainable development of environment. However, biological processes often require a previous acclimatization or special nutritional conditions, and many pollutants can form toxic or carcinogenic compounds which have a high bio-persistence. In the meantime, physical and chemical processes can generate second byproducts or transfer pollutants into other phases instead of destroying completely pollutants^3–5^. Therefore, advanced oxidation processes (AOPs) have been developed as a promising method for removing organic pollutants from aqueous environment, in which photocatalytic process using semiconductor is receiving much attention of researchers due to its advantages including environmental friendliness, no waste byproducts, complete degradation and mild operating conditions^6,7^. As the first photocatalyst discovered, titanium dioxide (TiO_2_) has been used widely to eliminate pollutants from water sources for many years^8^. However, the wide bandgap of TiO_2_ (3.2 eV) is only activated under UV region, which only occupies about 3–5% of solar light^9^. Therefore, visible light driven photocatalysts have been developed to use thoroughly the energy of solar light.

Among visible light driven photocatalyst, bismuth (Bi)-based photocatalysts have been attracting much attentions^10–12^, in which bismuth vanadate (BiVO_4_) has emerged as an effective photocatalyst due to its excellent properties including inexpensiveness, resistance to corrosion, high physicochemical stability and dispersibility^13,14^. Unfortunately, the photocatalytic activity of BiVO_4_ is restricted by the weak absorption and the poor migration of charged carriers^15,16^. In order to improve the photocatalytic activity of BiVO_4_, the combination of BiVO_4_ with appropriate materials is an effective way to enhance the specific surface area and the crystalline structure, leading to the increase of photocatalytic efficiency^17^. Among materials, silicon dioxide (SiO_2_) is known as an inexpensive material, well biocompatibility, large specific surface area, and easy functionalization; that can form p–n junction with BiVO_4_ to enhance the adsorptive ability and the separation of charged carriers^7,18–20^. In addition, graphene oxide (GO) is known as a 2D carbonaceous material which have large surface area (2630 m^2^/g), high conductivity and excellent mobility of charged carriers^19–23^. Therefore, GO is used as a favorable anchoring center to prevent the fast recombination of charged carriers, improving further the photocatalytic activity. As a result, it is expected that the combination of BiVO_4_, SiO_2_ and GO can generate a nanocomposite photocatalyst which have the best efficiency of photocatalytic activity.

Therefore, the objective of this work was to synthesize BiVO_4_/SiO_2_/GO nanocomposite by using solvothermal method. The sample was characterized by X-ray diffraction (XRD), scanning electron microscopy (SEM), and Brunauer–Emmett–Teller (BET) to clarify the morphology and crystalline structure. The photocatalytic activity was evaluated by the degradation of methylene blue (MB) and phenol under visible light irradiation. In addition, active species trapping test was also conducted to determine main species during the photocatalytic process of nanocomposite. Based on the obtained results, new insight of photocatalytic degradation of organic pollutant over BiVO_4_/SiO_2_/GO nanocomposite was found in the present work.

## Methodology

### Chemicals

Bismuth (III) nitrate pentahydrate (Bi(NO_3_)_3_·5H_2_O), ammonium metavanadate (NH_4_VO_3_), tetraethyl orthosilicate (SiC_8_H_20_O_4_), graphite flakes, sodium hydroxide (NaOH), sulfuric acid (H_2_SO_4_), potassium permanganate (KMnO_4_), hydrogen chloride (HCl), benzoquinone (C_6_H_4_O_2_), isopropyl alcohol (C_3_H_8_O), ammonium oxalate (C_2_H_8_N_2_O_4_), hydrogen peroxide (H_2_O_2_) were obtained from Sigma-Aldrich and were used as received without any further purification. The MB and phenol solution were prepared with DI water.

### Preparation of graphene oxide

In this work, graphene oxide was synthesized by Hummer’s method^22^ with a modification as follows: 1 g of graphite flakes and 0.5 g of NaNO_3_ was mixed in 100 mL of H_2_SO_4_ in an ice bath. Afterward, 6 g of KMnO_4_ was added carefully to the mixture to keep the temperature lower than 10 °C and stirred for 4 h. The ice bath was removed, then the mixture was heated at 35 °C for 12 h until it became a pasty brownish mixture. Subsequently, the mixture was cooled down to room temperature and added slowly with 200 mL of DI water. The obtained solution was reacted further with 5 mL of H_2_O_2_ to stop reaction. After centrifugation, resultant was washed with HCL (5%) for 3 times, then with ethanol and DI water for several times to achieve pH nature. Finally, the obtained sample was dried at 80 °C for 24 h.

### Preparation of nanocomposite

In the meantime, BiVO_4_-based nanocomposites were synthesized by using solvothermal method as follows: 5 mmol Bi(NO_3_)_3_·5H_2_O and 5 mmol NH_4_VO_3_ were stirred separately in 50 mL of ethanol for 30 min. These two solutions were then mixed together with the addition of 1 mL TEOS and 5 mL DI water for 1 h for the preparation of BiVO_4_/SiO_2_ core–shell. The pH of solution was adjusted to 5 by 2 M of NaOH solution. After that, the mixed solution was transferred into a Teflon-lined stainless steel and heated at 180 °C for 10 h. Finally, the resultant was washed with ethanol and DI water for several time, then dried at 80 °C for 24 h to achieve the final product. For the preparation of BiVO_4_/SiO_2_/GO nanocomposite, the obtained GO was sonicated in 20 mL of ethanol for 1 h in the first step. Secondly, 5 mmol Bi(NO_3_)_3_·5H_2_O and 5 mmol NH_4_VO_3_ were mixed in 100 mL of ethanol for 30 min, then followed by adding 1 mL of TEOS and 5 mL of DI water. In the third step, a calculated amount of GO solution was poured slowly into the above mixture. The fourth step is the pH adjustment of solution to 5 by NaOH of 2 M. Subsequently, the mixed solution was also heated at 180 °C for 10 h in the Teflon–lined stainless steel. After cooling, the BiVO_4_/SiO_2_/GO nanocomposites were obtained by washing with ethanol and Di water, which followed by drying at 80 °C for 24 h. In comparison, pure BiVO_4_ was also synthesized in the same conditions without the addition of SiO_2_ and GO.

### Characterizations

Crystal phase and structure of the as–synthesized samples were characterized by X–ray diffraction (XRD, Philips X'Pert MPD) using Cu Kα (λ = 1.54056 Å) radiation. The morphology was observed by transmission electron microscopy (TEM, JSM-2010, JEOL) and scanning electron microscopy (SEM, JEOL JSM-6335F). Brunauer–Emmett–Teller (BET) measurements (Adtosorb 1 MP, Quantachrome) were performed to determine the specific surface area of samples.

### Photocatalytic experiments

Typically, the photocatalytic experiments were performed at room temperature as follows: 0.05 g of photocatalyst was added into 50 mL of MB solution (3 ppm). The solution was stirred in dark condition for 30 min to achieve the adsorption–desorption equilibrium. After that, the solution was irradiated under visible light for 30 min by fluorescent lamp and the sample was collected every 10 min. The collected sample was centrifuged at 10,000 rpm for 15 min to remove the photocatalyst from dye solution. Finally, the concentration of dye solution was measured by UV–vis spectrophotometer (UV-6100, Mapada) at the wavelength of 664 nm to determine the removal efficiency. Additionally, isopropyl alcohol, benzoquinone and ammonium oxalate was used as scavenger for the hydroxyl radical, super oxide, and hole respectively to determine main species during the photocatalytic process of MB.

Besides, the photocatalytic degradation of phenol was also conducted in this work. In which, 50 mL of 20 ppm phenol solution and a small amount of H_2_O_2_ (35%) was stirred with 1 g/L of photocatalyst and for 30 min under dark condition. Afterwards, the mixed solution was irradiated under visible light for 360 min. The sample was collected at every 120 min followed by centrifugation, and then measured at 270 nm spectrophotometer (UV-6100, Mapada) to determine the removal efficiency.

## Results and discussions

### Characterizations

**Figure 1:**
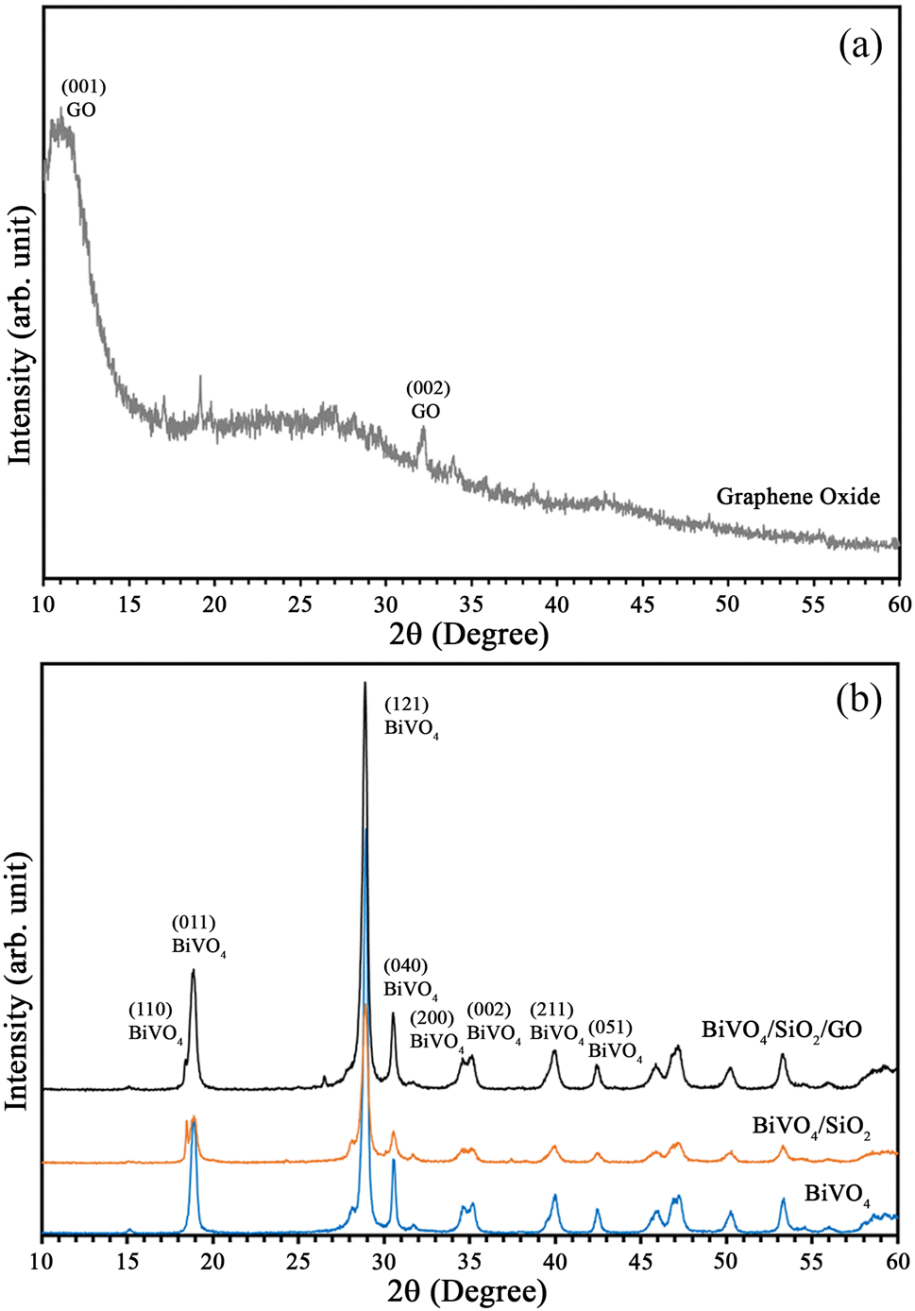
XRD patterns of (**a**) GO nanosheets and (**b**) BiVO_4_, BiVO4/SiO_2_ and BiVO_4_/SiO_2_/GO.

Figure 1 shows the XRD patterns of GO nanosheets, BiVO_4_ nanoparticles, BiVO_4_/SiO_2_ core–shell and BiVO_4_/SiO_2_/GO nanocomposite. In which, the XRD peak of graphite was mainly observed at around 36° of 2θ, however this peak was moved to 11° of 2θ after graphite was oxidized to graphene oxide. In the meantime, Fig. 1b shows that the as–prepared BiVO_4_ exhibited monoclinic phase corresponding to JCPDS No. 14-0688 which showed the highest photocatalytic activity^17^. Similarly, the diffraction peaks of BiVO_4_/SiO_2_ core–shell and BiVO_4_/SiO_2_/GO nanocomposite matched well with the monoclinic peaks of pure BiVO_4_ and no observation of other impurity peaks, this was due to the much lower content of SiO_2_ and GO in composite systems.

**Figure 2:**
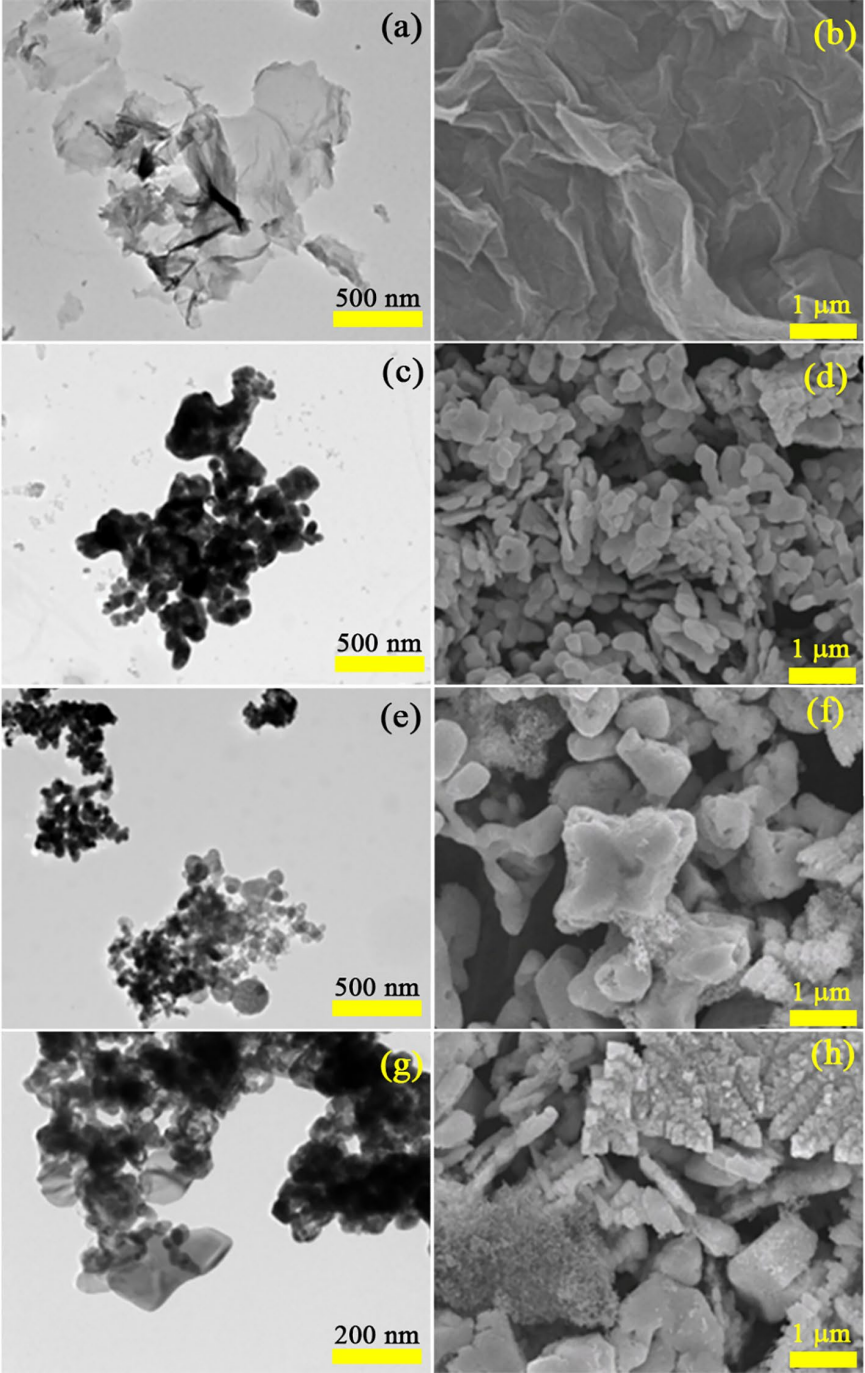
TEM and SEM images of GO (**a**,**b**), BiVO_4_ (**c**,**d**), BiVO_4_/SiO_2_ (**e**,**f**) and BiVO_4_/SiO_2_/GO (**g**,**h**).

The size and morphology of as–prepared samples were investigated via the SEM and TEM analysis, as shown in Fig. 2. It can be seen that the GO nanosheets were formed from thin sheets with wrinkled surface. In the meantime, the BiVO_4_ and BiVO_4_/SiO_2_ were the aggregation of amorphous nanoparticles with the size of several micrometers. Therefore, they were adhered uniformly on the GO sheets to form BiVO_4_/SiO_2_/GO nanocomposite by the reduction of GO to graphene from hydrothermal reaction and the facilitation from the functional groups of GO nanosheets.

### Photocatalytic activity

**Figure 3:**
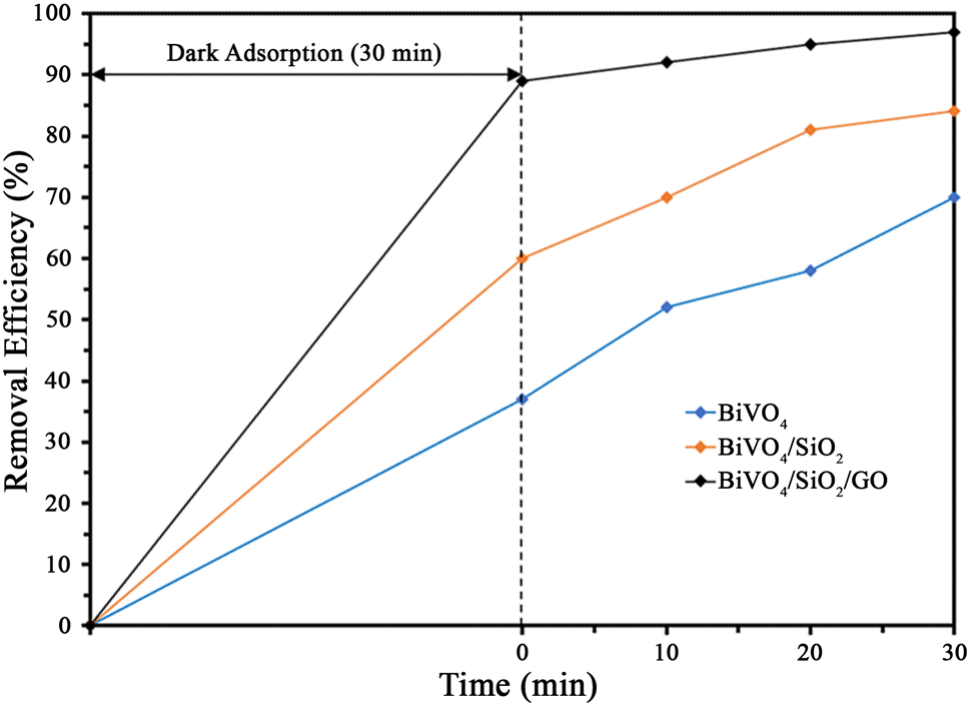
Photodegradation of MB over BiVO_4_, BiVO_4_/SiO_2_, BiVO_4_/SiO_2_/GO.

**Figure 4:**
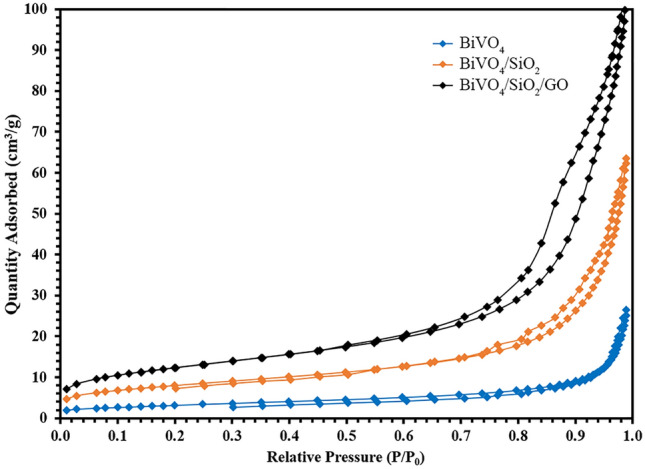
N_2_ Adsorption–desorption isotherm of as-prepared photocatalysts.

The photocatalytic activity of samples was evaluated by the degradation of organic pollutant including MB and phenol under visible light irradiation as shown in Figs. 3, 6. There were two mechanisms involving the pollutant removal efficiency; adsorption and photocatalytic degradation. From Fig. 3, all samples exhibited a high final efficiency for MB removal. In which, the BiVO_4_ nanoparticle adsorbed 35% of MB in the dark condition, and further degraded MB to 70% after 30 min of visible light irradiation. By coupling with SiO_2_, the adsorption of photocatalyst was increased to about 60%, leading to the final MB removal efficiency of 84%. In the meantime, the BiVO_4_/SiO_2_/GO nanocomposite showed the highest final MB removal efficiency of 94% with the high adsorption ability of 82%. The reason for excellent MB removal performance of BiVO_4_/SiO_2_/GO nanocomposite was that the specific surface area and the pore size were increased significantly by coupling with SiO_2_ and GO, as determined by BET characterization (Fig. 4 and Table 1).

**Table 1:** Surface properties of as–prepared photocatalysts.

Sample	Pore size (nm)	Specific surface area (m^2^/g)	Pore volume (cm^3^/g)
BiVO_4_/SiO_2_/GO	13.33	43.93	0.15
BiVO_4_/SiO_2_	12.29	28.47	0.09
BiVO_4_	12.11	11.13	0.03

Figure 4 shows that all samples exhibited N_2_ adsorption–desorption isotherms type IV and reserved H3 hysteresis loop^24,25^. This implied that the structures of all as-prepared samples were mesopores (width = 2–50 nm), which belonged to the average pore size diameter as reported in Table 1. The BET surface area and pore volume of BiVO_4_/SiO_2_/GO composite were 43.93 m^2^/g and 0.15 cm^3^/g; while those of single phase BiVO_4_ were 11.13 m^2^/g and 0.03 cm^3^/g, respectively. The quantity N_2_ adsorbed as well as other surface properties were found to increase as the SiO_2_ and GO were gradually added to BiVO_4_. The improvement of various surface properties of BiVO_4_/SiO_2_/GO were expected to benefit the pollutant to get adsorbed on the photocatalyst surface, which subsequently improved the photocatalytic process under light irradiation.

However, the adsorption ability was a key mechanism of photocatalyst as above discussion. The adsorption kinetic and isotherm of BiVO_4_/SiO_2_/GO nanocomposite was analyzed further, as in the supplement (Figures S1–S5, Tables S1, S2). The adsorption kinetic followed pseudo-second-order with a constant rate of 1.86 g/mg-min. The adsorption isotherm was defined as Freundlich isotherm with a Freundlich constant of 1.38 mg/g.

**Figure 5:**
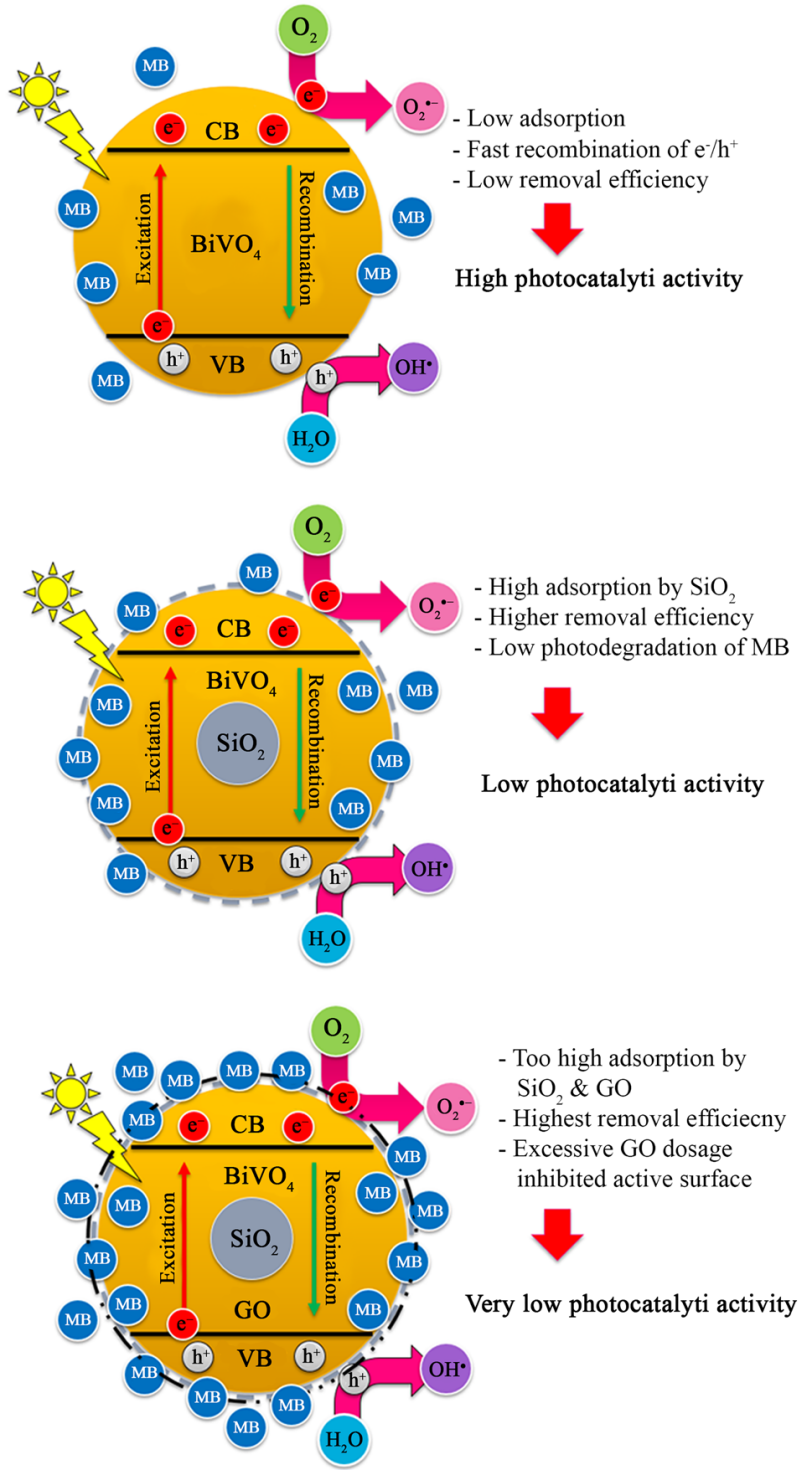
Summary of photodegradation of MB over BiVO_4_, BiVO4/SiO_2_ and BiVO_4_/SiO_2_/GO.

Although, the nanocomposite exhibited the highest final MB removal efficiency, it was found that the adsorption was extremely high instead of the photocatalytic degradation (Table S3). This meant that the pollutant was not degraded completely to CO_2_ and water by photocatalytic reactions, they were only trapped on the surface of SiO_2_ and GO. It demonstrated that the combination of the best ingredients (e.g., SiO_2_ and GO) could not make an excellent photocatalyst, instead it generated the excellent adsorbent in the present work. This is summarized in Fig. 5. According to the present work, the key mechanism on MB removal of BiVO_4_/SiO_2_ and BiVO_4_/SiO_2_/GO was adsorption, whereas that of BiVO_4_ was photocatalysis, as calculated in the supplement (Table S3).

**Figure 6:**
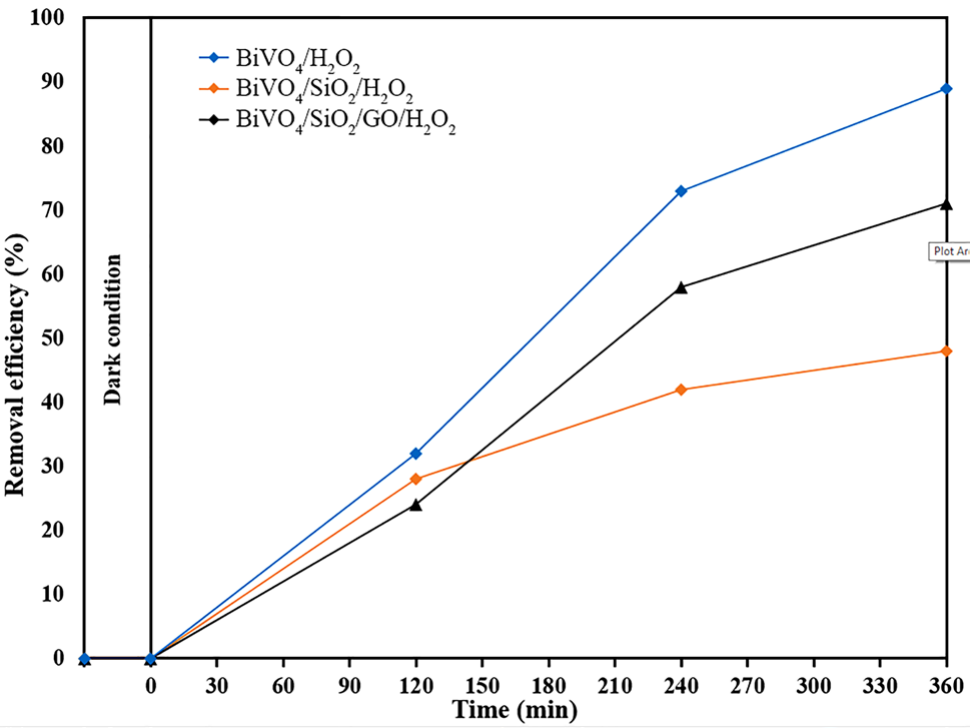
Photodegradation of phenol over BiVO_4_, BiVO_4_/SiO_2_ and BiVO_4_/SiO_2_/GO with the presence of H_2_O_2_.

In addition, phenol was chosen as a typical toxic pollutant to evaluate the photocatalytic activity of as–prepared samples as shown in Fig. 6. According to literature, phenol exists as phenoxide ions in aqueous environment, which has negative charge and high stability. Therefore, no adsorption of phenol was observed over all samples after 30 min of dark condition, and no photocatalytic degradation of phenol occurred under visible light irradiation of 6 h. In order to improve the photocatalytic activity for decomposing the highly stable structure of phenol, a small amount of H_2_O_2_ was added into the phenol solution as an assistance. This was because H_2_O_2_ could play an electron acceptor which decreased greatly the combination rate of electron–hole pairs, leading to the increase in hydroxyl radicals (listed in Eqs. 1–3) and then improving the photocatalytic activity. The BiVO_4_ nanoparticles showed the highest phenol removal efficiency of 92% after 6 h of visible light irradiation. In the meantime, the lowest efficiency was obtained in the BiVO_4_/SiO_2_ core–shell with 48% of phenol removal.

1 $${\text{H}}_{2}{{\text{O}}}_{2} +  {\text{e}}^{-} \, \to \, {\text{OH}}^{\cdot}  + {\text{OH}}^{-}$$

2 $${\text{OH}}^{\cdot} +  {\text{e}}^{-}  \to  {\text{OH}}^{-}$$

3 $${2}{\text{OH}}^{-}  +  {2}{\text{H}}^{+} \, \to \, {\text{2H}}_{2}{\text{O}}$$

**Figure 7:**
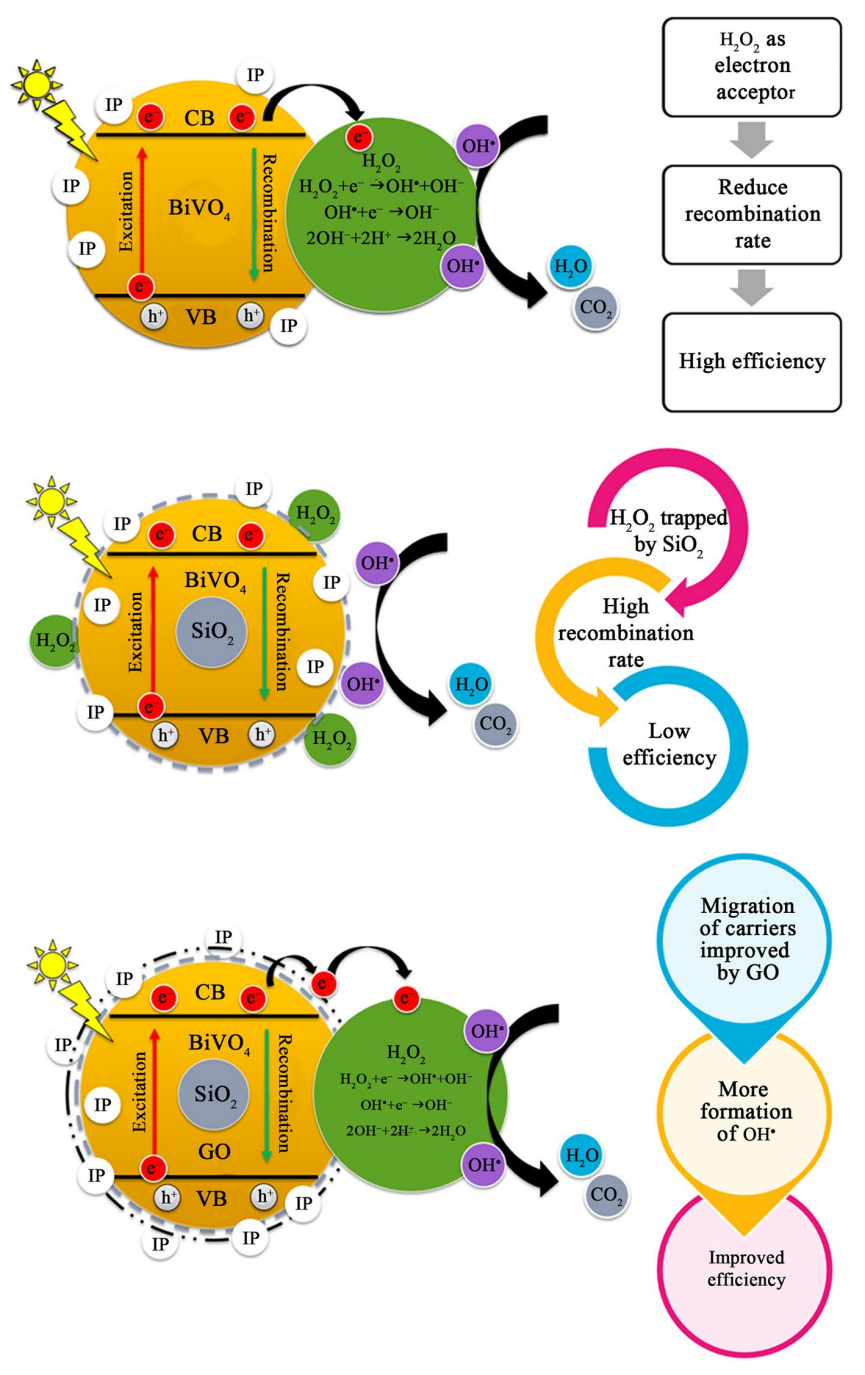
Photodegradation of phenol over BiVO_4_, BiVO_4_/SiO_2_, BiVO_4_/SiO_2_/GO with the presence of H_2_O_2_.

In the case of BiVO_4_/SiO_2_ core–shell, the reactions of H_2_O_2_ could be restricted by SiO_2_ layer, causing the difficult migration and the fast recombination of charged carriers. However, this inhibition was improved by the addition of GO nanosheets that enhanced the separation of electron–hole pairs. The phenol removal efficiency of BiVO_4_/SiO_2_/GO was improved to approximately 70%. This phenomenon is summarized in Fig. 7. Therefore, the best photocatalytic activity was occurred in BiVO_4_ nanoparticle, followed by BiVO_4_/SiO_2_/GO nanocomposite and BiVO_4_/SiO_2_ core–shell. However, the small amount of H_2_O_2_ addition was required to achieve the photocatalytic degradation of phenol. It can be seen that the types of pollutant were affected significantly on the key mechanism of BiVO_4_/SiO_2_/GO nanocomposite; the MB was removed mainly through adsorption due to the improving surface property of nanocomposite, whereas the phenol was removed through photocatalytic degradation due to its poor adsorption ability from phenoxide ions.

**Figure 8:**
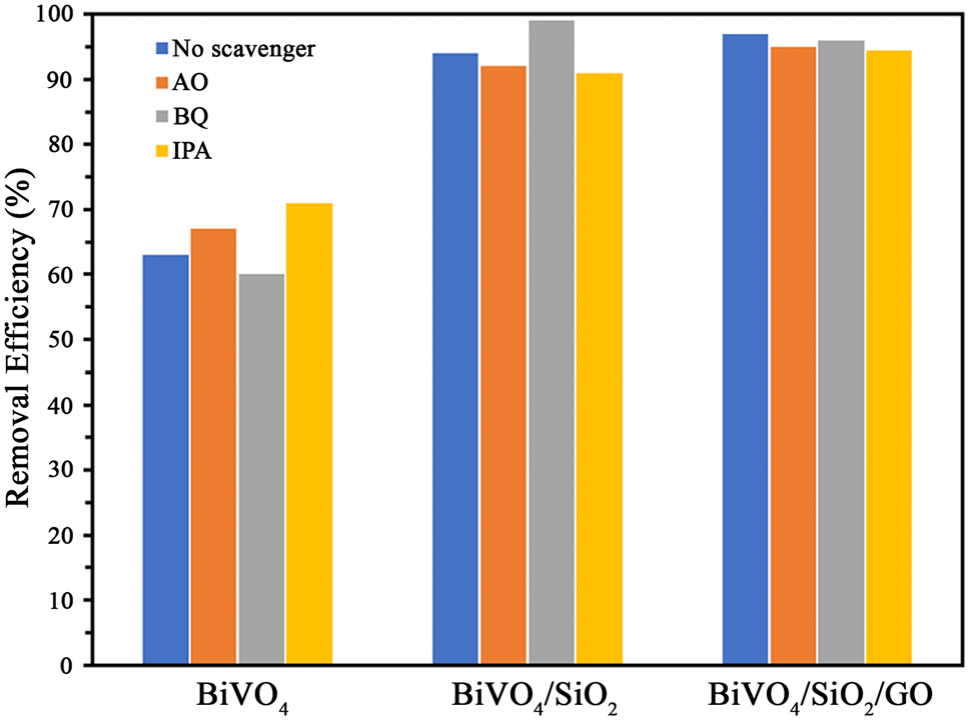
Effect of scavenger on the photodegradation of MB over BiVO_4_, BiVO_4_/SiO_2_ and BiVO_4_/SiO_2_/GO.

Due to the theoritical photocatalytic mechanism, the organic pollutant (i.e., phenol) was degraded by hydroxyl radical of photocatalyst and mineralized to CO_2_ and water. The organic concentration, represented in term of chemical oxygen demand (COD), was measured in the treated phenol solution to confirm the complete mineraliation and/or the occurance of intermediates. The initial COD concentration was 80 mg/L and it was decreased to approximately 46, 25 and 10 mg/L for BiVO_4_, BiVO_4_/SiO_2_ and BiVO_4_/SiO_2_/GO respectively in the 6 h-treated phenol solution. Therefore, the phenol molecule was degraded to small organic compounds, especially using BiVO_4_/SiO_2_/GO composite. The possible intermediates during phenol degradation were hydroquinone, benzoquinone, catechol and organic acid^26^. It should be noted that the existing H_2_O_2_ as interfering substrate could have a positive error effected on COD analysis^27^. It is important to be noted that the prove of present hydroxyradical is improtant as presented by several works^2,5,7,12,14,20,23,28^. In addition to the valuation of photocatalytic activity, the active species generated during the MB photocatalytic degradation were investigated by using scavenger as shown in Fig. 8.

**Figure 9:**
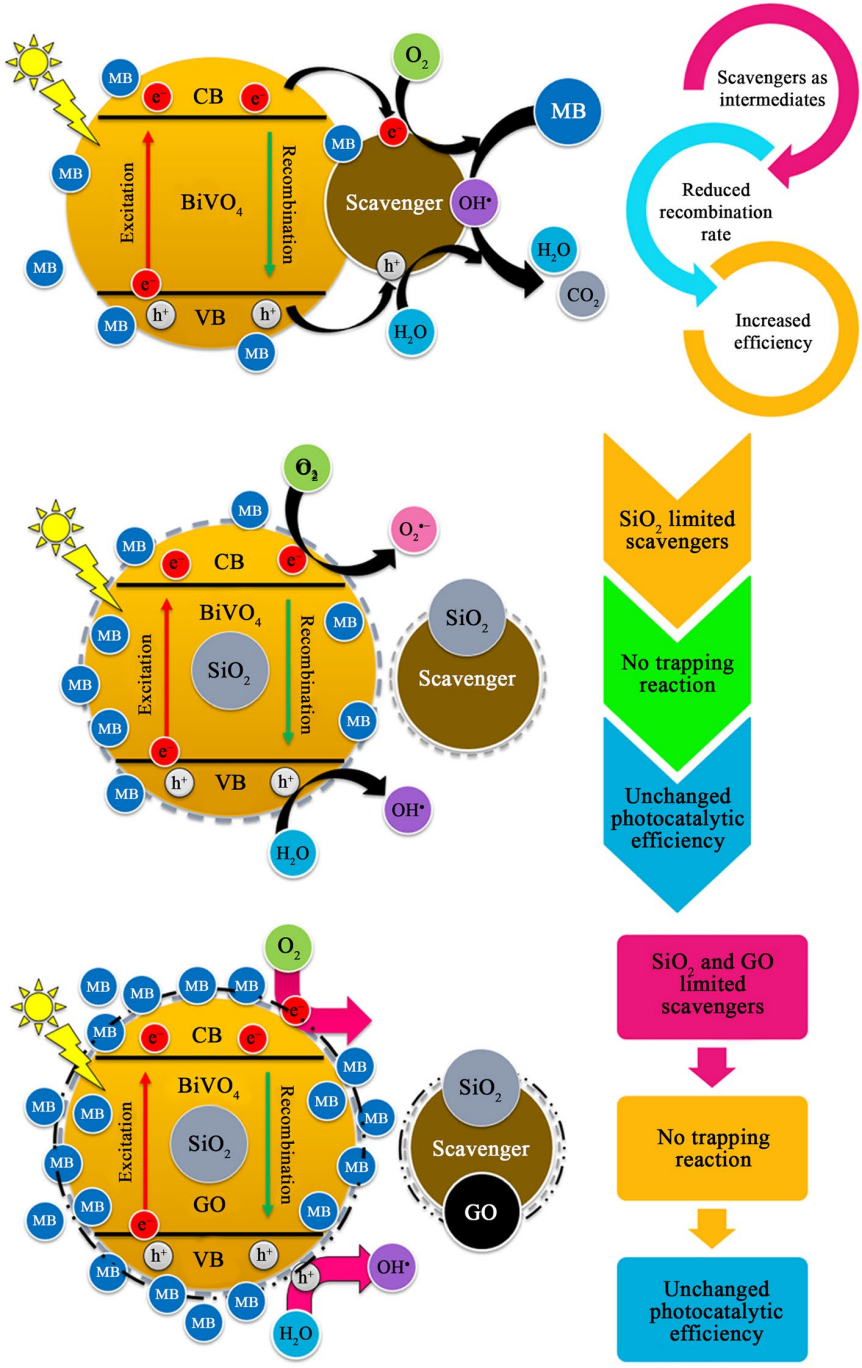
Photodegradation of MB over BiVO_4_, BiVO_4_/SiO_2_, BiVO_4_/SiO_2_/GO with the addition of scavenger.

The obtained results indicated that the photocatalytic degradation of BiVO_4_ was increased by the addition of AO and IPA. Unfortunately, this result was disagreement with previous studies because the recombination rate of electron–hole pairs of as–prepared BiVO_4_ was too fast, leading to less formation of hydroxyl radicals. Therefore, scavenger played as intermediate center which can reduce the recombination rate of electron–hole pairs, leading to the increase in photocatalytic degradation. In the meantime, not much change was observed in the photocatalytic process of BiVO_4_/SiO_2_ core–shell and BiVO_4_/SiO_2_/GO nanocomposite, because the scavenger can be adsorbed by SiO_2_ and GO layers, leading to the slight decrease in photocatalytic degradation. These results suggest that the scavenger test should be only used in the photocatalytic process which has much formation of active species. Figure 9 summarizes the photocatalytic degradation of MB over BiVO_4_ nanocomposite, BiVO_4_/SiO_2_ core–shell and BiVO_4_/SiO_2_/GO nanocomposite in the presence of scavenger.

## Conclusions

In the present work, the BiVO_4_/SiO_2_/GO nanocomposite was synthesized successfully by one–step solvothermal method. Nanocomposite was formed by the uniform adhesion of SiO_2_ and BiVO_4_ on GO nanosheets. Due to the low content of SiO_2_ and GO, the BiVO_4_/SiO_2_/GO nanocomposite exhibited the same XRD peaks of monoclinic BiVO_4_ without others contamination phase. The BiVO_4_/SiO_2_/GO showed the highest surface area of 43.93 m^2^/g, conversely the BiVO_4_ had only 11.13 m^2^/g. The BiVO_4_/SiO_2_/GO nanocomposite showed the higher adsorption ability rather than the photocatalytic degradation for MB removal. The enhancement of photocatalytic degradation was observed for phenol removal under H_2_O_2_ assistance. The BiVO_4_ nanoparticle still obtained the best photocatalytic ability than BiVO_4_/SiO_2_ core–shell and BiVO_4_/SiO_2_/GO nanocomposite. Although both SiO_2_ and GO were great property materials which able to improve the photocatalytic ability of BiVO_4_, the improper combination ratio caused the nanocomposite containing high adsorption ability and low photocatalytic degradation, as presented in this work. This is very important to be noted that the optimal combination ratio is essential to archive the greatest nanocomposite for photocatalytic application.

## Supplementary Information

Supplementary Information.
