# Supplementary material for: New insight into the photocatalytic degradation of organic pollutant over BiVO4/SiO2/GO nanocomposite
Source: Sci Rep. 2021 Feb 25;11:4620. doi: 10.1038/s41598-021-84323-5 (PMC7907200; doi:10.1038/s41598-021-84323-5)
Supplement: Supplementary file 1 — Supplementary Information. [file 41598_2021_84323_MOESM1_ESM.pdf]

Supplementary information: New insight into the photocatalytic degradation of organic pollutant over $BiVO_4/SiO_2$/GO nanocomposite

Dang Trung Tri Trinh, Duangdao Channei, Auppatham Nakaruk, Wilawan Khanitchaidecha

Corresponding Authors: wilawank1@gmail.com

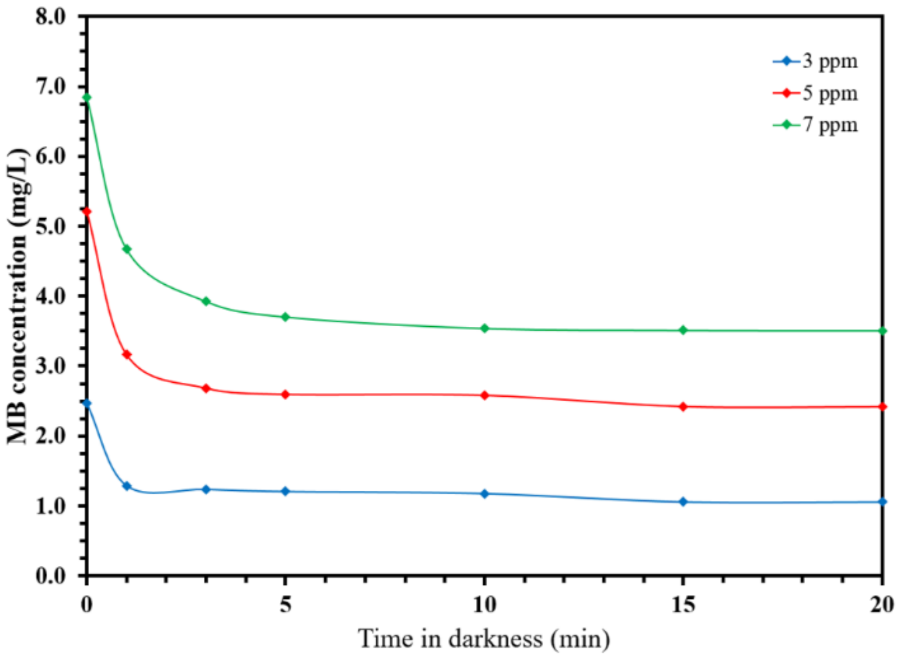

Figure S1. Reduction of MB concentration using $BiVO_4/SiO_2/GO$

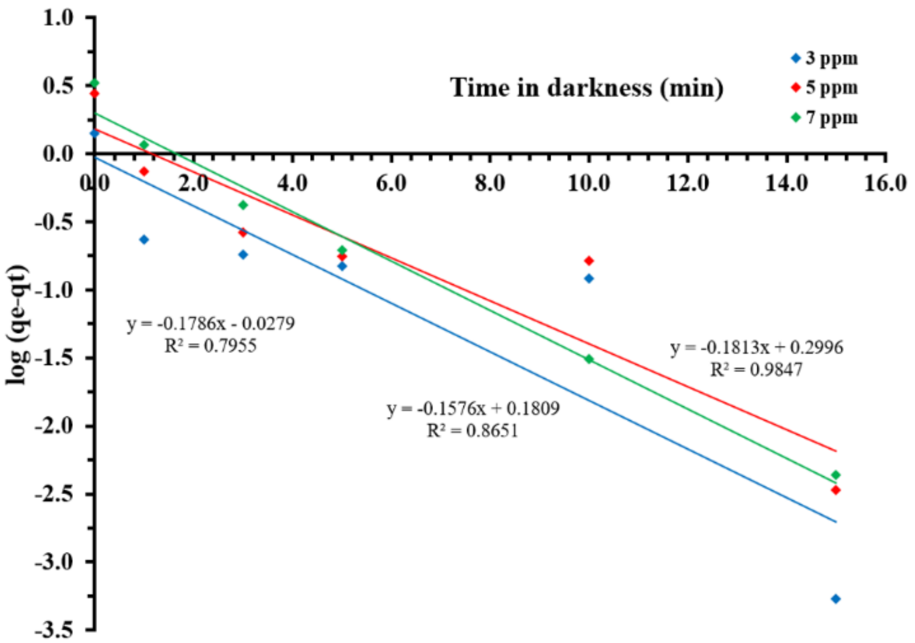

Figure S2. Adsorption kinetic of $BiVO_4/SiO_2/GO$ followed Pseudo-first-order

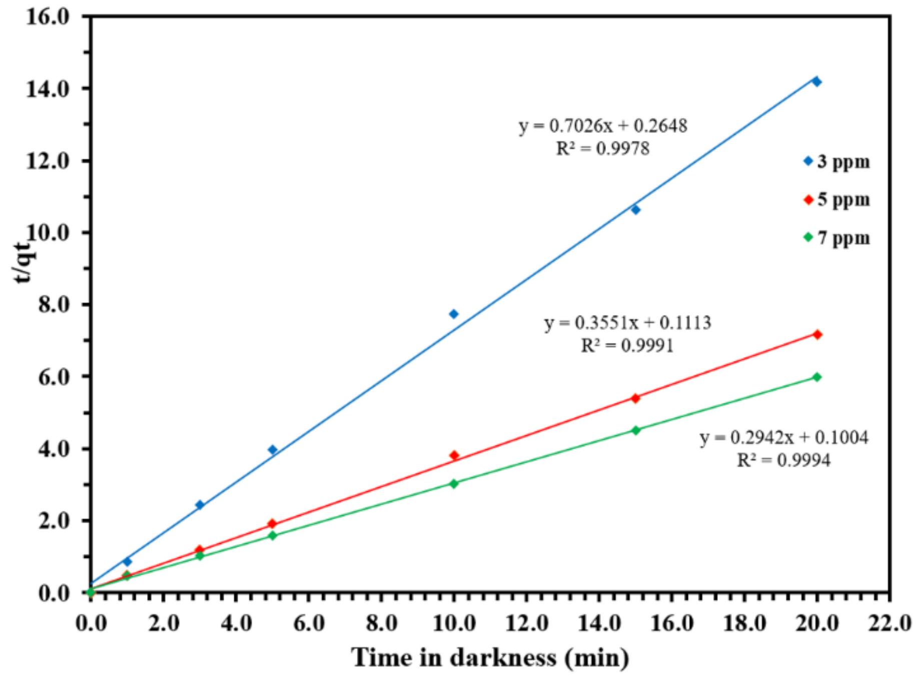

Figure S3. Adsorption kinetic of $BiVO_4/SiO_2$/GO followed Pseudo-second-order

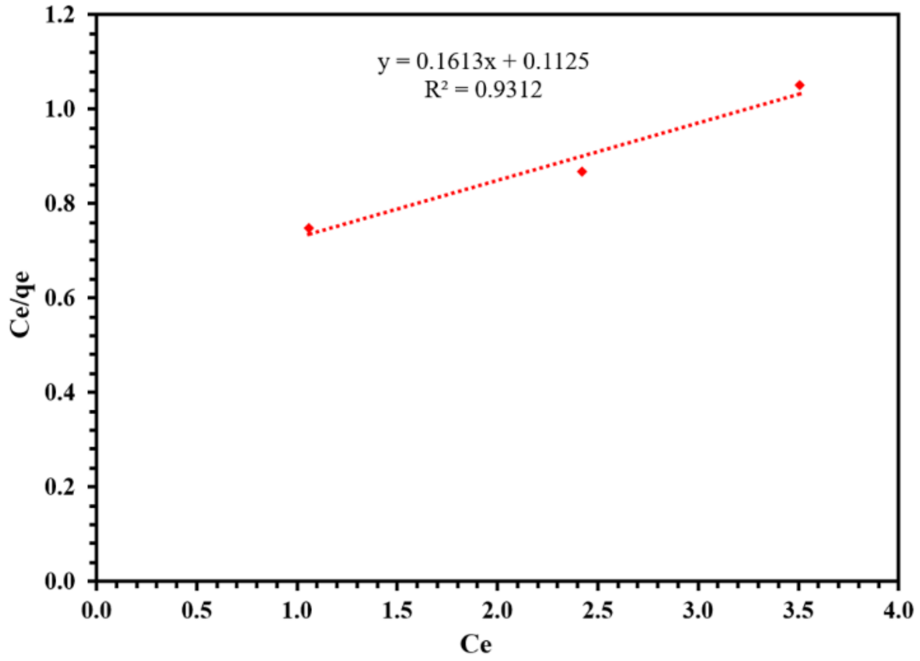

Figure S4. Adsorption isotherm of $BiVO_4/SiO_2$/GO followed Langmuir model

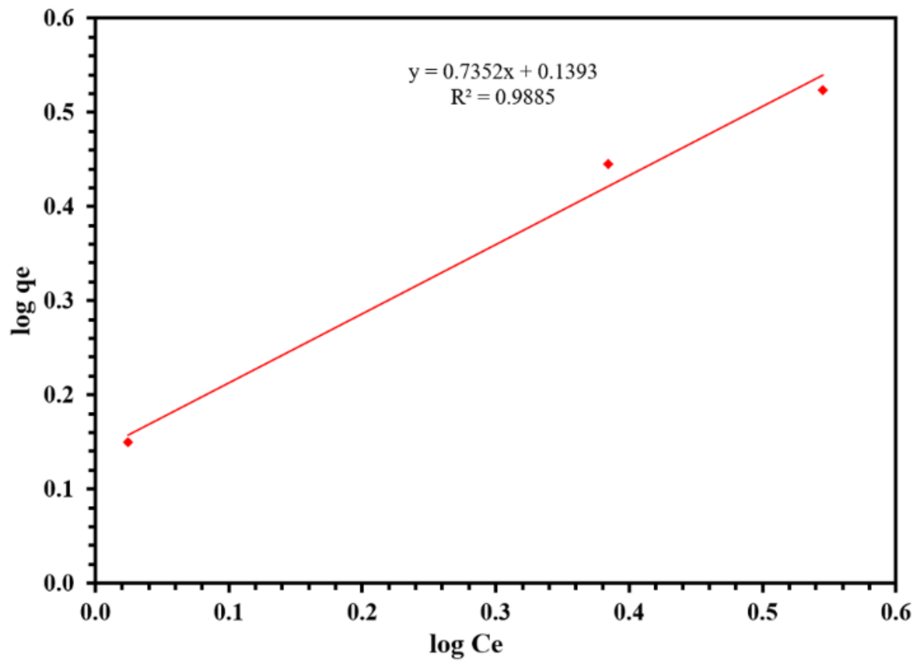

Figure S5. Adsorption isotherm of $BiVO_4/SiO_2/GO$ followed Freundlich model

Table S1. Adsorption kinetic of $BiVO_4/SiO_2/GO$ composite

**Initial MB** (ppm)	**Pseudo-first-order**			**Pseudo-second-order**		
	q_e (mg/g)	K (min^{-1})	R^2	q_e (mg/g)	K2 (g/mg-min)	R^2
3	1.066	0.411316	0.7955	1.4233	1.8642	0.9978
5	1.5167	0.362953	0.8651	2.8161	1.1329	0.9991
7	1.9934	0.417534	0.9847	3.3990	0.8621	0.9994

Table S2. Adsorption isotherm of $BiVO_4/SiO_2/GO$

**Langmuir constants**	Q^0	6.20
	b	1.43
	R^2	0.9312
**Freundlich constants**	n	1.360
	K_F	1.378
	R^2	0.9985

Table S3. MB removal by adsorption and photocatalysis of nanocomposites

**Sample**	**Adsorption efficiency (%) (in the darkness, 30 min)**	**Photodegradation efficiency (%) (in visible light irradiation, 30 min)**
$BiVO_4$	37	33
$BiVO_4$-SiO_2	60	24
$BiVO_4$-SiO_2-GO	89	8
